# Caffeine Promotes Conversion of Palmitic Acid to Palmitoleic Acid by Inducing Expression of *fat-5* in *Caenorhabditis elegans* and *scd1* in Mice

**DOI:** 10.3389/fphar.2018.00321

**Published:** 2018-04-06

**Authors:** Xiaocui Du, Qin Huang, Yun Guan, Ming Lv, Xiaofang He, Chongye Fang, Xuanjun Wang, Jun Sheng

**Affiliations:** ^1^Key Laboratory of Pu-erh Tea Science, Ministry of Education, Yunnan Agricultural University, Kunming, China; ^2^Tea Research Center of Yunnan, Kunming, China; ^3^College of Food Science and Technology, Yunnan Agricultural University, Kunming, China; ^4^College of Science, Yunnan Agricultural University, Kunming, China; ^5^State Key Laboratory for Conservation and Utilization of Bio-Resources in Yunnan, Kunming, China

**Keywords:** caffeine, *Caenorhabditis elegans*, Δ9 desaturases, *fat-5*, fatty acid composition, 3T3L1, *scd1*, *pgc-1*α

## Abstract

The synthesis and metabolism of fatty acids in an organism is related to many biological processes and is involved in several diseases. The effects of caffeine on fatty acid synthesis and fat storage in *Caenorhabditis elegans* and mice were studied. After 6 h of food deprivation, adult *C. elegans* were treated with 0.1 mg/mL caffeine for 24 h. Quantitative reverse-transcription polymerase chain reaction showed that, among all the genes involved in fat accumulation, the mRNA expression of *fat-5* in caffeine-treated *C. elegans* was significantly higher than that of controls, whereas *fat-6* and *fat-7* displayed no significant difference. Gas chromatography-mass spectrometry was used to verify the fatty acid composition of *C. elegans*. Results showed that the ratio of palmitoleic acid (16:1) to that of palmitic acid (16:0) was higher in the caffeine-treated group. Several mutant strains, including those involved in the insulin-like growth factor-1, dopamine, and serotonin pathways, and nuclear hormone receptors (*nhrs*), were used to assess their necessity to the effects of caffeine. We found that *mdt-15* was essential for the effects of caffeine, which was independent of *nhr-49* and *nhr*-80. Caffeine may increase *fat-5* expression by acting on *mdt-15*. In high fat diet (HFD), but not in normal diet (ND) mice, caffeine induced expression of *scd1* in both subcutaneous and epididymal white adipose tissue, which was consistent with the palmitoleic/palmitic ratio results by gas chromatograph analysis. In mature adipocytes, caffeine treatment induced both mRNA and protein expression of *scd1* and *pgc-1*α. Overall, our results provided a possible mechanism on how caffeine modulates metabolism homeostasis *in vivo*.

## Introduction

Caffeine is a psychoactive substance used worldwide. Caffeine is present in tea, coffee, chocolate, and soda beverages (Temple, 2009). The primary mechanism of action of caffeine *in vivo* is antagonism of adenosine receptors (Nehlig, 1999). Due to the correlation between adenosine receptors and the dopamine system, caffeine exerts various physiological, behavioral, and psychological effects (Kudlacek et al., 2003). Besides its effects on the nervous system, caffeine consumption has been reported to reduce blood glucose levels in mice suffering from diabetes mellitus (DM) (Yamauchi et al., 2010). In fact, habitual intake of caffeine or coffee has been reported to correlate closely with the metabolism of glucose and fat, as reviewed by Heckman et al. (2010). Caffeine has been shown to reduce body weight in animals (Zheng et al., 2004) and, in some studies, reduce weight gain in humans (Lopez-Garcia et al., 2006). However, several studies have shown no increase in weight loss induced by the addition of caffeine to a low-calorie diet in humans (Kovacs et al., 2004). In the present study, we aimed to discover the effects of caffeine upon fatty acid synthesis and fat accumulation, and validated these effects in a model organism employed to investigate energy metabolism: *Caenorhabditis elegans*.

The nematode *C. elegans* has become a popular model for exploring the genetic basis of fatty acid synthesis and regulation of fat storage (Mullaney and Ashrafi, 2009). Several lipid metabolic pathways found in mammals are well conserved in *C. elegans* (Van Gilst et al., 2005), including pathways for fatty acid synthesis, elongation, and desaturation, and mitochondrial and peroxisomal β-oxidation of fatty acids (Watts, 2009). As reviewed by Watts (2009), three major pathways influence adiposity in *C. elegans*: serotonin, insulin, and transforming growth factor β (TGF-β). Four transcription factors and the transcriptional mediator *mdt-15* contribute to fat storage. In the present study, we found that caffeine promoted fat storage under fasting conditions by increasing expression of *fat-5*, which was independent of nuclear hormone receptor (NHR)-49 and NHR-80, but the presence of *mdt-15* was necessary.

Stearoyl-coenzyme A desaturase (SCD) is a key enzyme that catalyzes the formation of monounsaturated fatty acids (MUFAs) at the ninth-position carbon chain of saturated fatty acids (SFAs). The first cloned subtype of *scd1* gene is *scd1* in mice, which is expressed widely in various tissues and organs, especially in adipose tissue and the liver (Ntambi et al., 1988). *Pgc-1*α is a transcription co-activator that regulates numerous genes involved in lipid and energy metabolism (Settembre et al., 2013; Cheng et al., 2017).

Studies have shown that *scd1, fat-5, fat-6*, and *fat-7* are homologous in mice, and that *pgc1*α and *mdt15* have similar roles in mice and nematodes, respectively. We have also studied the changes in expression of *scd1* and *pgc1*α in white adipose tissue and 3T3L1 adipocytes after caffeine treatment (Brock et al., 2007; Taubert et al., 2008).

To elucidate the effects of caffeine in mammals, we also investigated the effects of caffeine on the synthesis and metabolism of fatty acids in the white adipose tissue of mice. SCD1 is a rate-limiting enzyme responsible for MUFA synthesis, and introduces a double bond in the *cis*-delta-9 position of some saturated fatty acetyl coenzyme A molecules (Enoch et al., 1976). Excessive intake of SFAs results in high levels of cholesterol, triglycerides, and low-density-lipoprotein cholesterol in blood, which can increase the risk of arterial stenosis, atherosclerosis, and coronary heart disease. Unsaturated fatty acids (e.g., MUFAs, polyunsaturated fatty acids) are of considerable benefit to human health. For example, they can regulate lipids levels in blood. High lipid levels in blood are the main causes of hypertension, arteriosclerosis, heart disease, cerebral thrombosis, and stroke.

In the present study, to explore the impact of caffeine on fatty acid conversions, we treated mice and adipocytes with caffeine. The results showed that, after treatment with caffeine, *scd1* expression was upregulated. The ratio of palmitoleic acid (16:1)/palmitic acid (16:0) in epididymal adipose tissue and subcutaneous adipose tissue of mice was upregulated in HFD fed mice. That is, mice fed caffeine promoted the conversion of SFAs to MUFAs.

## Materials and Methods

### *Caenorhabditis elegans* Strains and Growth Conditions

The *C. elegans* strains N2-Bristol (wild-type, WT), *mdt-15* (XA7702), *fat-5* (BX107), *dop-1* (LX636), *dop-2* (LX702), *mod-1* (MT9668), *daf-3* (CB1376), *daf-2* (DR1568), *age-1*(TJ1052), *daf-16* (CF1038), *NHR-49* (RB1716), *NHR-80* (bx165), and *sbp-1* (CE541) were provided by the Caenorhabditis Genetics Center (University of Minnesota, Minneapolis, MN, United States). All strains were cultured at 20°C.

Unless stated otherwise, *C. elegans* were grown on nematode growth media (NGM) plates with the OP50 strain of *Escherichia coli* (with or without caffeine) as a food source. Caffeine was added directly to the OP50 food source to feed *C. elegans*. For synchronization, *C. elegans* in the gravid stage were transferred onto plates containing OP50 so that their eggs could be laid. When a sufficient number of eggs had been laid, adult *C. elegans* were moved, and the eggs laid by them were of the same stage.

For analyses of mRNA and triglycerides, as well as gas chromatography (GC), synchronized *C. elegans* were grown on caffeine-free NGM plates for 72 h. Then, they were transferred onto NGM plates with or without caffeine and cultured for 24 h. After 6 h of food deprivation in M9 buffer on bacteria-free NGM plates, *C. elegans* were harvested and washed with phosphate-buffered saline or M9 buffer, and then fixed or crushed for use.

### Quantitative Reverse-Transcription Polymerase Chain Reaction (RT-PCR)

Starved or fed *C. elegans* were collected, and RNA was prepared using TRIzol^®^ Reagent (Invitrogen, Carlsbad, CA, United States). After quantification, 1 mg of total RNA was used in a reverse-transcription reaction with SuperScript^®^ III (Invitrogen) to generate cDNA.

The 3T3L1 preadipocytes (2 × 10^5^ cells/well) were seeded in 60-mm culture dishes into adipocytes and then treated with caffeine (0, 50, 100, 200, 400, or 800 mg/mL) for 24 h. Total RNA was extracted from samples of subcutaneous fat and epididymal adipose tissue (50 mg) using TransZol Up (TransGen Biotech, Beijing, China) according to manufacturer’s protocols. Reverse transcription was undertaken using a PrimeScript^TM^ RT Reagent kit with gDNA Eraser (TaKaRa Biotechnology, Kusatsu, Japan) according to manufacturer’s instructions. Quantitative RT-PCR (qRT-PCR) was done using a SYBR Premix Ex Taq^TM^ II kit (Tli RNase H Plus; TaKaRa Biotechnology) and the results were determined using a 7900HT Fast RT-PCR system (Applied Biosystems, Foster City, CA, United States). Data were calculated using the comparative 2^-ΔΔC_t_^ method, and all values were normalized to the mRNA level of the endogenous gene 36b4.

The PCR mixture consisted of 0.3 mM primers, cDNA, ROX Reference, and SYBR green mix (Platinum^®^ SYBR^®^ Green qPCR SuperMix-UDG; Invitrogen). qRT-PCR was run on an ABI 7900 system (Applied Biosystems, Foster City, CA, United States), and the level of each mRNA was normalized to that of the corresponding act-1. The primer sequences (Generay Biotech, Shanghai, China) are provided in **Table 1**.

**Table 1:** Primer sequences of genes for RT-PCR.

Primer name	Forward (5′–3′)	Reverse (5′–3′)
*act-1*	GCTGGACGTGATCTTACTGATTACC	GTAGCAGAGCTTCTCCTTGATGTC
*fat-5*	CGGCCGCCCTCTTCCGTTAC	TGGCTGCCATCCGACCCAGT
*fat-6*	TCAACAGCGCTGCTCACTAT	TTCGACTGGGGTAATTGAGG
*fat-7*	CAACAGCGCTGCTCACTATT	CACCAACGGCTACAACTGTG
*pgc-1*α	CGGAAATCATATCCAACCAG	TGAGGACCGCTAGCAAGTTTG
*scd1*	TTCCCTCCTGCAAGCTCTAC	CAGAGCGCTGGTCATGTAGT
*36b4*	TCGCTTTCTGGAGGGTGT	TTCAGTAAGTGGGAAGGTGT

### Measurement of Composition of Fatty Acids in *C. elegans*

Analyses of the fatty acid composition of *C. elegans* were done as described previously (Watts and Browse, 2002). Briefly, 200 adult *C. elegans* were collected with M9 buffer and washed three times. After centrifugation, M9 buffer was removed with a pipette and replaced with 1 mL 2.5% H_2_SO_4_ in methanol to extract fatty acids from tissues and then to carry out their transmethylation. Samples were capped and incubated for 1 h at 80°C. After the addition of 0.2 mL hexane and 1.5 mL water, fatty acid methyl esters were extracted into the hexane layer by shaking and centrifugation of the tubes at low speed. Aliquots (1 μL) of the organic phase were analyzed by GC using a gas chromatograph (QP2010 series; Shimadzu, Kyoto, Japan) equipped with a RTX-5MS column (30 × 0.25 mm). Mass conditions: EI source (70 ev), dual-filament, scanning range (50–500 m/z), scan interval 1.0 s. Helium was the carrier gas (injected at 1.4 mL/min), and a flame ionization detector was employed. The gas chromatograph was programmed at an initial temperature of 120°C for 1 min, followed by an increase of 10°C/min to 190°C, followed by an increase of 2°C/min to 200°C. The mass spectra of the peaks identified as palmitic acid, palmitoleic acid, oleic acid, and stearic acid matched the spectra presented by W. W. Christie on the lipid library website^1^.

### Animal Models and Experimental Protocols

All experimental procedures were carried out in accordance with the guidelines set by the Committee for Care and Use of Laboratory Animals of Yunnan Agricultural University (Kunming, China). These procedures were approved by the Animal Experiments Ethics Committee of Yunnan Agricultural University.

A total of 36 male specific-pathogen-free C57BL/6J (6-week-old) mice (Cavens Laboratory Animals, Nanjing, China) were housed in the Key Laboratory of Pu-er Tea Science, Ministry of Education, Yunnan Agricultural University. Mice were housed in polypropylene cages with sterile paddy husks. They were maintained under a controlled environment (humidity = 50–60%; ambient temperature = 24 ± 1°C; 12-h light–dark cycle) and allowed to adapt to their environment for 1 week.

Mice were assigned randomly to two groups. One group was fed a chow diet (CD; *n* = 18) and another was fed a high-fat diet (HFD; 60% energy from fat; *n* = 18; D12492; Research Diets, New Brunswick, NJ, United States) for 12 weeks to establish HFD-model mice. Mice had free access to water and were weighed once a week.

The CD mice maintained on a chow diet were divided into two groups (*n* = 9). The chow diet with caffeine group had free access to water containing 0.5 mg/mL (1.5 mg/day for a 30-g mouse, relative to 300 mg/day for a 60-kg person) caffeine (referred to our previous study Chong-Ye et al., 2015; Cai et al., 2016) in sterilized tap water during the entire feeding period. The chow diet group received vehicle.

The HFD-induced mice maintained on a HFD diet were divided into two groups (*n* = 9). The HFD with caffeine group had free access to water containing 0.5 mg caffeine in 1 mL sterilized tap water during the entire feeding period. The HFD group received vehicle.

Food and water intake was recorded once a week. The mice were fed 12 weeks and sacrificed without fasting. Samples of subcutaneous fat and epididymal adipose tissue were collected and stored at -80°C.

### Cell Culture

The 3T3-L1 preadipocytes (American Type Culture Collection, Manassas, VA, United States) were maintained in Dulbecco’s Modified Eagle’s Medium (DMEM; 4 mM L-glutamine, 4500 mg/L glucose, sodium pyruvate; Thermo Scientific, Waltham, MA, United States) with 10% fetal bovine calf serum (FBS; Biological Industries Israel Beit Haemek, Beit Haemek, Israel) in a humidified atmosphere with 5% CO_2_ at 37°C.

### Differentiation of 3T3-L1 Preadipocytes Into Mature Adipocytes

The 3T3-L1 preadipocytes were cultured until they reached 100% confluence under normal culture. After 48 h, the culture medium was changed to DMEM containing 5 μg/mL insulin, 70.5 μg/mL 3-isobutyl-1-methylxanthine, 0.096 μg/mL dexamethasone, and 0.47 μg/mL rosiglitazone (MDI Induction Media; Sigma–Aldrich, Saint Louis, MO, United States) and supplemented with 10% FBS for 3 days. Subsequently, the cells were incubated for 2 days with MDI Induction Media and Insulin Media (DMEM supplemented with 10% FBS and 5 μg/mL insulin) at 1:1. Then, the cells were incubated for 3 days with Insulin Media. Finally, DMEM supplemented with 10% FBS was used for 2 days.

### Protein Preparation and Western Blotting

The 3T3L1 preadipocytes (2 × 10^5^ cells/well) were seeded in 60-mm culture dishes into adipocytes and then treated with caffeine (0, 50, 100, 200, 400, and 800 mg/mL) for 24 h. Western blotting was done as described previously (Cai et al., 2016). Briefly, cell lysates were prepared from cultured cells, subcutaneous adipose tissue, and epididymal adipose tissue using RIPA buffer (Solarbio, Beijing, China) according to manufacturer’s protocols. Cell extracts and tissue extracts were normalized to determine protein concentrations by the bicinchoninic acid method. Proteins were separated by sodium dodecyl sulfate–polyacrylamide gel electrophoresis and then transferred to polyvinylidene difluoride (PVDF) membranes (Millipore, Bedford, MA, United States). After gentle washing, blocking, and incubation with the primary antibody (Anti-PGC-1α antibody was purchased from Millipore, Bedford, MA, United States; anti-SCD1 antibody was from Cell Signaling Technology, Danvers, MA, United States; anti-β-tubulin antibody was purchased from Proteintech, Chicago, IL, United States. Dilution of anti-β-tubulin antibody, anti-SCD1 antibody, and anti-PGC-1α antibody were 1:1000. Secondary antibody of anti-β-tubulin antibody and anti-PGC-1α antibody were anti-mouse IgG; secondary antibody of anti-SCD1 antibody was anti-rabbit IgG. Anti-mouse IgG and anti-rabbit IgG were purchased from R&D Systems, Minneapolis, MN, United States. Dilution of anti-mouse IgG and anti-rabbit IgG were 1:5000), and PVDF membranes were incubated with the appropriate horseradish peroxidase-conjugated secondary antibody. Protein bands were detected using a Pro-light Horseradish Peroxidase Chemiluminescence kit (Tiangen Biotech, Beijing, China). Images were acquired with a FluorChem^TM^ E system (ProteinSimple, Santa Clara, CA, United States).

### Measurement of the Fatty Acid Composition of Adipose Tissue in Mice

A 900-μL portion of trichloromethane methanol (2:1) was used to extract 100 μg subcutaneous fat or epididymal fat. Adipose tissue was broken down using a tissue homogenizer (TissueLyser II, Qiagen Inc., Stanford, VA, United States) and a 1-mL extract added overnight at 4°C; this step was repeated. Then, fatty acid methyl esters were obtained by the boron trifluoride method (GB/T 17376-2008/ISO 5509:2000). Fatty acid methyl esters were analyzed with a gas chromatograph (7890A; Agilent Technologies, Palo Alto, CA, United States) equipped with an automatic column injector, a capillary column (30 m × 250 μm × 0.25 μm), and a hydrogen flame ionization detector.

### Statistical Analyses

**FIGURE 1:**
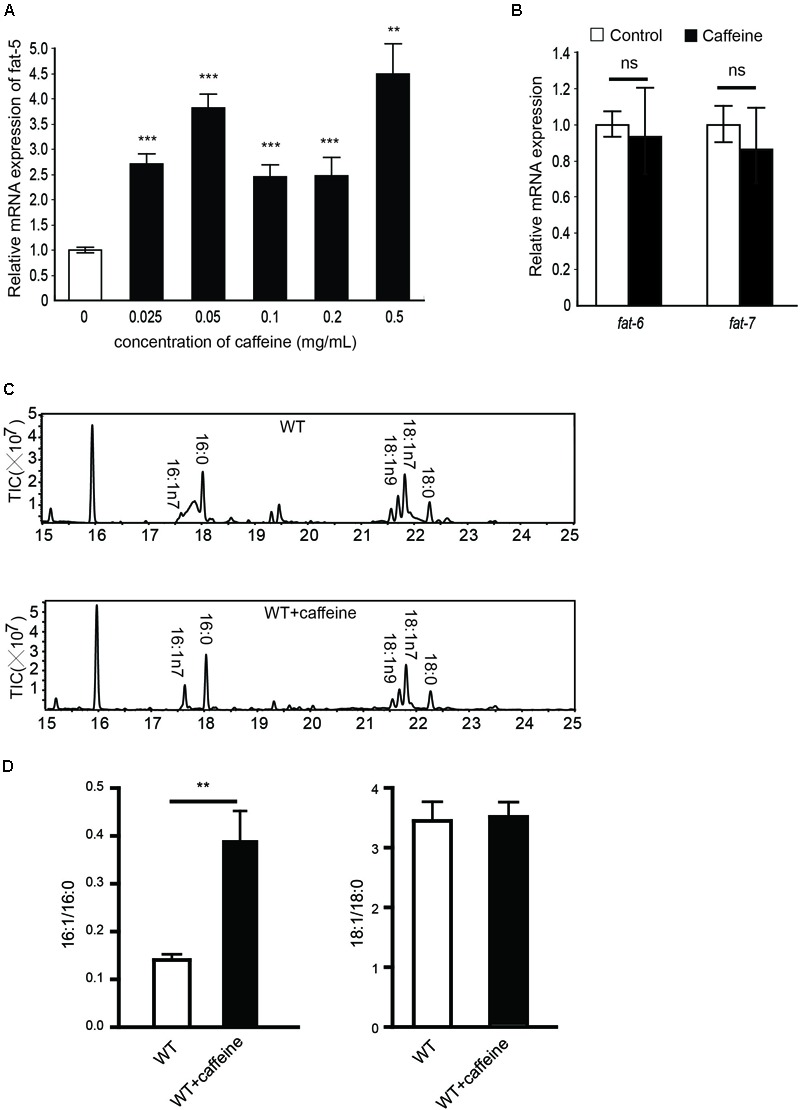
mRNA and fatty acid compositions of caffeine-treated worms. **(A)** 0.025, 0.05, 0.1, 0.2, 0.5 mg/mL caffeine all increased mRNA expression of *fat-5*, 0.1 mg/mL caffeine increased *fat-5* expression approximately 2.5-fold; **(B)** 0.1 mg/mL caffeine did not affect mRNA expression of *fat-6* or *fat-7*; **(C,D)** Results of fatty acid contents by GC analysis. Treatment with 0.1 mg/mL caffeine elevated the 16:1/16:0 ratio (three repeats, *p* = 0.0054). No significant distinction was observed in the 18:1/18:0 ratio. It was analyzed using Student’s *t*-test. The data are described as mean ± SEM; ^∗∗^*p* < 0.01; ^∗∗∗^*p* < 0.001.

**FIGURE 2:**
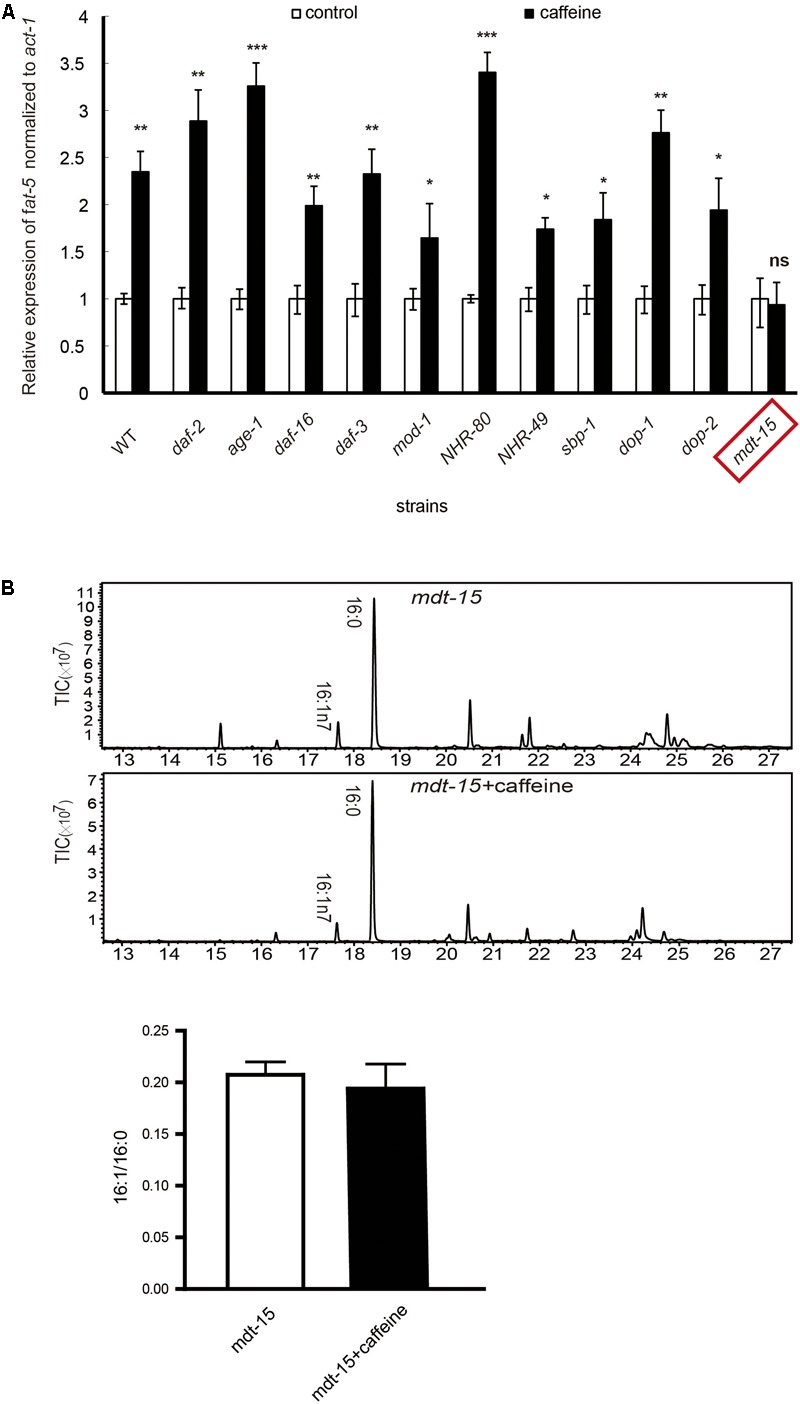
mRNA expression and fatty acid compositions of worms. **(A)** The extent of increased expression in wild-type was about 2.5-fold. In all other mutant strains, the extent ranged from 1.5- to 3.5-fold. *fat-5* mRNA expression was not significantly increased in *mdt-15* mutant worms. **(B)** Fatty acid content results by GC-MS analysis. All assays were repeated at least three times. **(B)** It was analyzed using Student’s *t*-test. The data are described as mean ± SEM; ^∗^*p* < 0.05; ^∗∗^*p* < 0.01; ^∗∗∗^*p* < 0.001.

**FIGURE 3:**
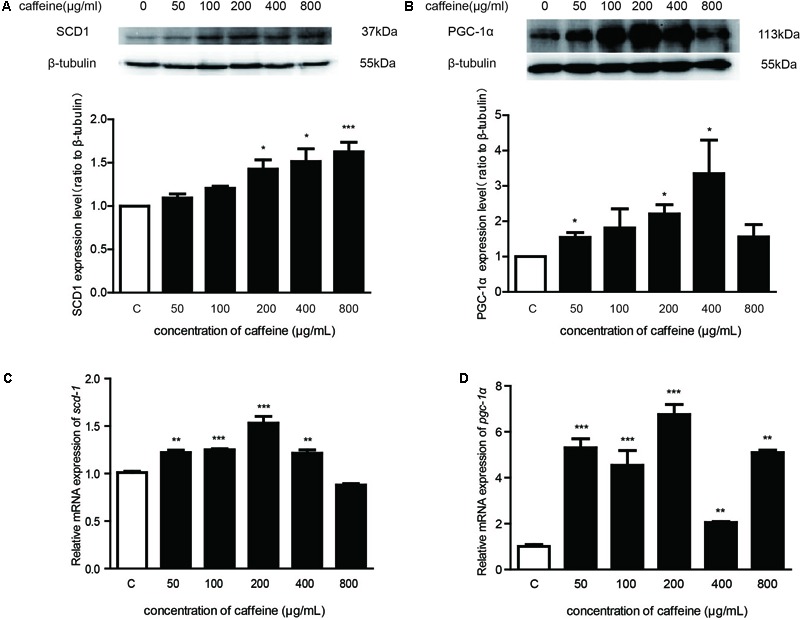
Effects of caffeine on scd1 and pgc-1α protein and mRNA expression in mature adipocytes. **(A)** The protein expression of key genes involved in formation of fatty acids in mature adipocytes. **(B)** The mRNA expression of key genes involved in formation of monounsaturated fatty acids in mature adipocytes (*n* = 4). The data are described as mean ± SEM; ^∗^*p* < 0.05; ^∗∗^*p* < 0.01; ^∗∗∗^*p* < 0.001.

**FIGURE 4:**
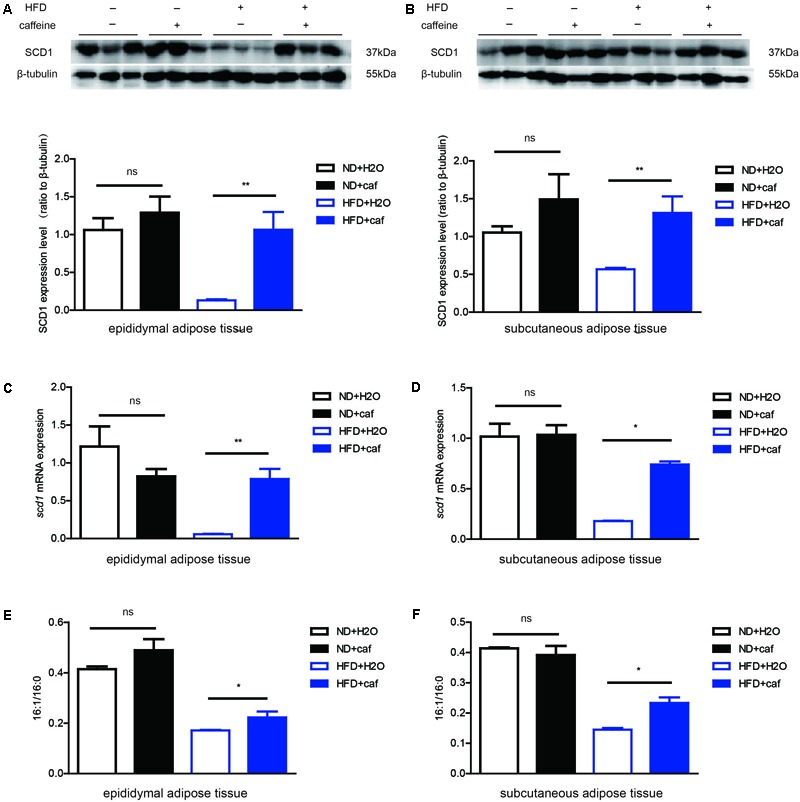
Caffeine treatment increased the formation of monounsaturated fatty acids in epididymal adipose tissue and subcutaneous adipose tissue in mice. **(A,B)** The protein expression of scd1 in epididymal adipose tissue and subcutaneous adipose tissue (*n* = 9). **(C,D)** The mRNA expression of scd1 in epididymal adipose tissue and subcutaneous adipose tissue (*n* = 9). **(E,F)** Fatty acid contents in epididymal adipose tissue and subcutaneous adipose tissue by GC analysis. 16:1/16:0 ratio (*n* = 9). The data are described as mean ± SEM; ^∗^*p* < 0.05; ^∗∗^*p* < 0.01.

Statistical analysis was performed using GraphPad (version 5.0). Data are the mean ± SEM. Unless otherwise noted, one-way ANOVA followed with a Dunnett’s Multiple Comparison Test was used to evaluate the significance of differences among groups in **Figures 1–3**. Two-way ANOVA followed with a Bonferroni’s post-tests was used to evaluate the significance of differences among groups in **Figure 4**. *p* < 0.05 was considered significant.

## Results

### Caffeine Increased the mRNA Expression and Activity of *fat-5*, but Not That of *fat-6* or *fat-7*

*Caenorhabditis elegans* encodes three Δ9 desaturases: *fat-5, fat-6, and fat-7*. Due to its crucial role in the regulation of MUFA synthesis, we first examined the effect of caffeine intake on mRNA expression of Δ9 desaturases to investigate how MUFA synthesis promotes the effects of caffeine.

After 6 h of food deprivation, the RNA of *C. elegans* was isolated and qRT-PCR used to determine expression of these three Δ9 desaturases. Caffeine increased *fat-5* expression in a dose-dependent manner (**Figure 1A**). We found that 0.1 mg/mL caffeine increased *fat-5* expression by 2.5-fold, whereas 0.1 mg/mL caffeine did not affect the expression of *fat-6* or *fat-7* significantly (**Figure 1B**).

The *fat-5* metabolizes palmitic acid (16:0) to produce palmitoleic acid (16:1n7). The fatty acid composition of caffeine-treated *C. elegans* was analyzed using GC-mass spectrometry to determine *fat-5* activity. The palmitoleic-acid content of the caffeine-treated group was significantly higher than that of the non-treated group (**Figure 1C**), and the palmitoleic acid (16:1)/palmitic acid (16:0) ratio was increased by approximately twofold by caffeine (**Figure 1D**). No obvious difference in the oleic acid (18:1)/stearic acid (18:0) ratio between the caffeine-treated group and non-treated group was observed. fat-6 and fat-7 catalyze the conversion of stearic acid (18:0) into oleic acid (18:1). Hence, this result was consistent with the mRNA expression of *fat-6* and *fat-7*. Taken together, these results suggested that caffeine increased the mRNA expression and activity of *fat-5*, but not that of *fat-6* or *fat-7*.

### The Effect of Caffeine Was Not Dependent on Insulin-Like Growth Factor (IGF)-1, Serotonin, Dopamine, or TGF-β Pathways, but Was Dependent on *mdt-15*

We further studied how caffeine increases the mRNA expression of *fat-5*. The three major pathways involved in fat storage (IGF-1, TGF-β, serotonin) were examined. In addition, the role of three transcription factors (NHR-49, NHR-80, sbp-1) on the effects of caffeine was studied by qRT-PCR.

Relevant mutant strains (*daf-2, age-1, daf-16, daf-3, mod-1, nhr-49, nhr-80, sbp-1*) were treated with 0.1 mg/mL caffeine, and mRNA expression of *fat-5* was detected by RT-PCR. Usually, the actions of caffeine *in vivo* need the activation of dopamine receptors. Hence, how caffeine promotes *fat-5* expression in fasting *C. elegans* was also examined in *dop-1* and *dop-2* mutant strains.

The mRNA expression of *fat-5* was increased by approximately 2.5-fold by caffeine in WT *C. elegans* and, in all these mutant strains, caffeine treatment led to a 1.5- to 3.5-fold increase in *fat-5* expression (**Figure 2A**). These results suggested that mutation of these genes did not have an impact on the effects of caffeine. That is, the *fat-5* expression-promoting effect of caffeine did not need the presence of these genes. The only exception to this rule was the *mdt-15* mutant strain: a significant increase in *fat-5* expression was not observed, which suggested that the effect of caffeine needed the presence of *mdt-15*. To further identify the crucial role of *mdt-15*, we analyzed the fatty acid composition of the *mdt-15* mutant strain. Caffeine did not increase the palmitoleic acid (16:1)/palmitic acid (16:0) ratio when *mdt-15* was mutant (**Figure 2B**), which implied that *mdt-15* was necessary for the effects of caffeine.

### Caffeine Treatment Induced SCD1 and PGC-1α Expression in Cultured Mature Adipocytes

In the experiments detailed above, we demonstrated that *fat-5* and *mdt-15* had roles in the effects of caffeine in *C. elegans*. Also, studies have shown that in mice, *scd1* is a homolog of *fat-5*, and that *pgc1*α has a similar role to that of *mdt-15.* Therefore, we detected whether *scd1* and *pgc1*α expressions were induced by caffeine in adipocytes.

Mature adipocytes were treated with various concentrations of caffeine. Results showed that, after treatment with caffeine, SCD1 and PGC-1α expressions in mature adipocytes were upregulated significantly in a concentration-dependent manner (**Figures 3A,B**). We examined the expression of *scd1* mRNA and *pgc-1*α mRNA in mature adipocytes, and found it to be increased significantly (*p* < 0.05) (**Figures 3C,D**). Taken together, these results suggested that caffeine treatment induced the expression of *scd1* and *pgc-1*α in adipocytes.

### Caffeine Increased the Formation of Palmitoleic Acid in the Epididymal Fat and Subcutaneous Fat of Mice

C57BL/6J mice were fed a normal chow diet (ND) and HFD, respectively. After 12 weeks, epididymal adipose tissue and subcutaneous adipose tissue were isolated, and the protein and RNA extracted.

In both epididymal adipose tissue and subcutaneous adipose tissue, HFD resulted in a reduction of expression of SCD1, which might be attributed to the low level of de novo lipogenesis inhibited by excessive fat intake. Caffeine treatment induced protein (**Figures 4A,B**) and mRNA (**Figures 4C,D**) expression of SCD1 in both white adipose tissues in HFD mice, but not in white adipose tissues in ND mice. Fatty acid composition analysis results showed that in HFD mice, caffeine elevated 16:1/16:0 ratio in epididymal adipose tissue and subcutaneous adipose tissue (**Figures 4E,F**).

Overall, these results demonstrated that caffeine treatment increased the conversion of palmitic acid (16:0) to palmitoleic acid (16:1) in white adipose tissues in HFD-fed mice.

## Discussion

**FIGURE 5:**
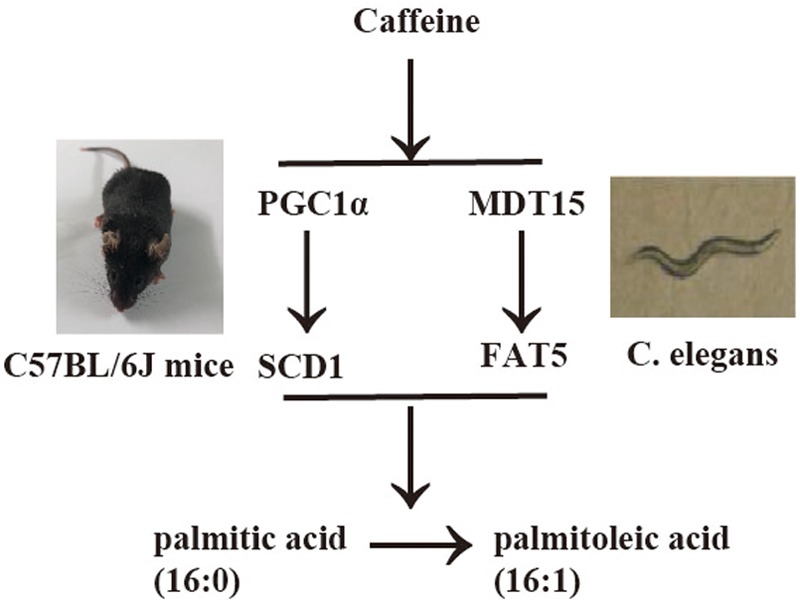
Synoptic figure summarizing caffeine’s effects on conversion of palmitic acid into palmitoleic acid. Caffeine induces expression of Δ9 desaturases (*fat-5* in *C. elegans* or *scd-1* in mice) by modulating *mdt-15* in nematode *C. elegans* or PGC-1α in mice, which play similar roles in these two models. The image of *C. elegans* was cited from Watts and Browse (2002).

In the present study, we demonstrated that caffeine promoted the conversion of palmitic acid (16:0) to palmitoleic acid (16:1) by inducing expression of Δ9 desaturases in *C. elegans* (**Figures 1, 2**). Key metabolic pathways and their regulators in *C. elegans* are evolutionarily conserved and should be broadly informative (Mullaney and Ashrafi, 2009). Our results suggested that caffeine induced *scd-1* expression in white adipose tissue and cultured adipocytes, and promoted palmitic acid converting into palmitoleic acid in white adipose tissues (**Figures 3, 4**), which were in accordance with the results in *C. elegans* (**Figure 5**).

The *fat-5* is homolog of mammalian *scd-1*, which also catalyzes the conversion of palmitic acid to palmitoleic acid (Flowers and Ntambi, 2009). *scd-1* plays a key part in the regulation of lipid biosynthesis and carbohydrate metabolism. *scd-1* deficiency leads to leanness as well as an increased metabolic rate and insulin sensitivity in mice (Ntambi et al., 2002). Interestingly, administration of T2DM drugs functioning as the peroxisome proliferator-activated receptor-g agonist glitazone leads to increased mRNA expression and activity of *scd-1* in humans (Risérus et al., 2005; Yao-Borengasser et al., 2008). Activation of *scd-1* has been reported to promote lipid accumulation in mice (Ntambi et al., 2002) and caffeine induced *scd-1* expression but is believed to inhibit lipid accumulation (Zheng et al., 2004). This contradiction remains to be explained, which needs further investigations.

We have reported that caffeine lowers glucose levels in plasma in BALB/c mice (Chong-Ye et al., 2015) and that caffeine can alleviate nonalcoholic fatty liver disease (NAFLD) in HFD-fed C57BL/6J mice (Cai et al., 2016). It has been reported that C16:1n7-palmitoleate acts as an adipose tissue-derived lipid hormone that strongly stimulates the action of insulin in muscle and suppresses hepatic steatosis (Cao et al., 2008). Our results imply that: (i) the expression and activity of *scd-1* promotes the effects of caffeine; (ii) these effects may be related to its plasma-glucose lowering and anti-NAFLD actions; (iii) caffeine may potent treatment for these effects. In summary, our results provided a possible mechanism on how caffeine modulates glucose metabolism homeostasis.

The *mdt-15* is a subunit of the *C. elegans–*mediator complex; it integrates regulation of fatty acid metabolism by *nhr-49*-dependent and *nhr-49*-independent pathways (Taubert et al., 2006). In the present study, we found that mutations of *nhr-49, nhr-80*, or *sbp-1* did not affect the actions of caffeine, so it seems that caffeine regulated the metabolism of fatty acids by an *nhr-49, nhr-80, or sbp-1-*independent pathway. It has been demonstrated that *nhr-49:mdt-15* functions overlaps those of *ppar*α*:pgc-1* and/or *hnf4*α*:pgc-1* even though *C. elegans* lacks the orthologs of *pgc-1* proteins (Taubert et al., 2006). Caffeine induced *pgc-1*α expression in the adipose tissue of mice (**Figure 3**), which further suggested that *pgc-1*α had a similar role to *mdt-15* in *C. elegans* and was involved in MUFA synthesis.

The regulatory mechanism of *mdt-15* has not been demonstrated fully in *C. elegans*, so the pathway through which caffeine acts on *mdt-15* is not clear. According to our results, several receptors (e.g., those for IGF-1, serotonin, or dopamine) are not needed for the effects of caffeine. Caffeine may enter cells and target *mdt-15* directly to regulate fatty acid metabolism and lipid accumulation. The exact mechanism underlying caffeine’s impacts on *mdt-15* in *C. elegans* or *pgc-1*α in mice needs further study.

## Conclusion

Caffeine promotes conversion of palmitic acid to palmitoleic acid by inducing expression of *fat-5* in *Caenorhabditis elegans* and *scd1* in mice, and PGC-1α (MDT-15) might be involved in this process.

## Author Contributions

JS, XW, and CF conceived and designed the experiments. XD, QH, YG, ML, and XH performed the experiments. XD and QH analyzed the data. JS, XW, and CF contributed to reagents/materials/analysis tools. XD, QH, and CF wrote the manuscript. All authors read and approved the final manuscript.

## Conflict of Interest Statement

The authors declare that the research was conducted in the absence of any commercial or financial relationships that could be construed as a potential conflict of interest.

## Footnotes

1 http://lipidlibrary.aocs.org/

## References

[B1] Brock T. J. Browse J. Watts J. L. (2007). Fatty acid desaturation and the regulation of adiposity in *Caenorhabditis elegans*. *Genetics* 176 865–875. 10.1534/genetics.107.071860 17435249 PMC1894614

[B2] Cai X. Fang C. Hayashi S. Hao S. Zhao M. Tsutsui H. (2016). Pu-erh tea extract ameliorates high-fat diet-induced nonalcoholic steatohepatitis and insulin resistance by modulating hepatic IL-6/STAT3 signaling in mice. *J. Gastroenterol.* 51 819–829. 10.1007/s00535-015-1154-0 26794005

[B3] Cao H. Gerhold K. Mayers J. R. Wiest M. M. Watkins S. M. Hotamisligil G. S. (2008). Identification of a lipokine, a lipid hormone linking adipose tissue to systemic metabolism. *Cell* 134 933–944. 10.1016/j.cell.2008.07.048 18805087 PMC2728618

[B4] Cheng L. Zhu Y. Han H. Zhang Q. Cui K. Shen H. (2017). MicroRNA-148a deficiency promotes hepatic lipid metabolism and hepatocarcinogenesis in mice. *Cell Death Dis.* 8:e2916. 10.1038/cddis.2017.309 28703810 PMC5550856

[B5] Chong-Ye F. Xuan-Jun W. Ye-Wei H. Shu-Mei H. Jun S. (2015). Caffeine is responsible for the bloodglucose-lowering effects of green tea and Puer tea extractsin BALB/c mice. *Chin. J. Nat. Med.* 13 595–601. 10.3724/SP.J.1009.2015.00595 26253492

[B6] Enoch H. G. Catala A. Strittmatter P. (1976). Mechanism of rat liver microsomal stearyl-CoA desaturase. *J. Biol. Chem.* 251 5095–5103. 8453

[B7] Flowers M. T. Ntambi J. M. (2009). Stearoyl-CoA desaturase and its relation to high-carbohydrate diets and obesity. *Biochim. Biophys. Acta* 1791 85–91. 10.1016/j.bbalip.2008.12.011 19166967 PMC2649790

[B8] Heckman M. A. Weil J. Gonzalez de Mejia E. (2010). Caffeine (1, 3, 7-trimethylxanthine) in foods: a comprehensive review on consumption, functionality, safety, and regulatory matters. *J. Food Sci.* 75 R77–R87. 10.1111/j.1750-3841.2010.01561.x 20492310

[B9] Kovacs E. M. Lejeune M. P. Nijs I. Westerterp-Plantenga M. S. (2004). Effects of green tea on weight maintenance after body-weight loss. *Br. J. Nutr.* 91 431–437. 10.1079/BJN20041061 15005829

[B10] Kudlacek O. Just H. Korkhov V. M. Vartian N. Klinger M. Pankevych H. (2003). The human D2 dopamine receptor synergizes with the A2A adenosine receptor to stimulate adenylyl cyclase in PC12 cells. *Neuropsychopharmacology* 28 1317–1327. 10.1038/sj.npp.1300181 12784121

[B11] Lopez-Garcia E. van Dam R. M. Rajpathak S. Willett W. C. Manson J. E. Hu F. B. (2006). Changes in caffeine intake and long-term weight change in men and women. *Am. J. Clin. Nutr.* 83 674–680. 10.1093/ajcn.83.3.674 16522916

[B12] Mullaney B. C. Ashrafi K. (2009). *C. elegans* fat storage and metabolic regulation. *Biochim. Biophys. Acta* 1791 474–478. 10.1016/j.bbalip.2008.12.013 19168149 PMC2772880

[B13] Nehlig A. (1999). Are we dependent upon coffee and caffeine? A review on human and animal data. *Neurosci. Biobehav. Rev.* 23 563–576. 10.1016/S0149-7634(98)00050-5 10073894

[B14] Ntambi J. M. Buhrow S. A. Kaestner K. H. Christy$ R. J. Sibley E. Kelly T. J. (1988). Differentiation-induced gene expression in 3T3-Ll preadipocytes. *J. Biol. Chem.* 263 17291–17300. 2903162

[B15] Ntambi J. M. Miyazaki M. Stoehr J. P. Lan H. Kendziorski C. M. Yandell B. S. (2002). Loss of stearoyl–CoA desaturase-1 function protects mice against adiposity. *Proc. Natl. Acad. Sci. U.S.A.* 99 11482–11486. 10.1073/pnas.132384699 12177411 PMC123282

[B16] Risérus U. Tan G. D. Fielding B. A. Neville M. J. Currie J. Savage D. B. (2005). Rosiglitazone increases indexes of stearoyl-CoA desaturase activity in humans link to insulin sensitization and the role of dominant-negative mutation in peroxisome proliferator–activated receptor-γ. *Diabetes Metab. Res. Rev.* 54 1379–1384. 10.2337/diabetes.54.5.1379 15855323

[B17] Settembre C. De Cegli R. Mansueto G. Saha P. K. Vetrini F. Visvikis O. (2013). TFEB controls cellular lipid metabolism through a starvation-induced autoregulatory loop. *Nat. Cell Biol.* 15 647–658. 10.1038/ncb2718 23604321 PMC3699877

[B18] Taubert S. Hansen M. Van Gilst M. R. Cooper S. B. Yamamoto K. R. (2008). The Mediator subunit MDT-15 confers metabolic adaptation to ingested material. *PLoS Genet.* 4:e1000021. 10.1371/journal.pgen.1000021 18454197 PMC2265483

[B19] Taubert S. Van Gilst M. R. Hansen M. Yamamoto K. R. (2006). A Mediator subunit, MDT-15, integrates regulation of fatty acid metabolism by NHR-49-dependent and -independent pathways in *C. elegans*. *Genes Dev.* 20 1137–1149. 10.1101/gad.1395406 16651656 PMC1472473

[B20] Temple J. L. (2009). Caffeine use in children: what we know, what we have left to learn, and why we should worry. *Neurosci. Biobehav. Rev.* 33 793–806. 10.1016/j.neubiorev.2009.01.001 19428492 PMC2699625

[B21] Van Gilst M. R. Hadjivassiliou H. Jolly A. Yamamoto K. R. (2005). Nuclear hormone receptor NHR-49 controls fat consumption and fatty acid composition in *C. elegans*. *PLoS Biol.* 3:e53. 10.1371/journal.pbio.0030053 15719061 PMC547972

[B22] Watts J. L. (2009). Fat synthesis and adiposity regulation in *Caenorhabditis elegans*. *Trends Endocrinol. Metab.* 20 58–65. 10.1016/j.tem.2008.11.002 19181539 PMC2665873

[B23] Watts J. L. Browse J. (2002). Genetic dissection of polyunsaturated fatty acid synthesis in *Caenorhabditis elegans*. *Proc. Natl. Acad. Sci. U.S.A.* 99 5854–5859. 10.1073/pnas.092064799 11972048 PMC122866

[B24] Yamauchi R. Kobayashi M. Matsuda Y. Ojika M. Shigeoka S. Yamamoto Y. (2010). Coffee and caffeine ameliorate hyperglycemia, fatty liver, and inflammatory adipocytokine expression in spontaneously diabetic KK-Ay mice. *J. Agric. Food Chem.* 58 5597–5603. 10.1021/jf904062c 20405946

[B25] Yao-Borengasser A. Rassouli N. Varma V. Bodles A. M. Rasouli N. Unal R. (2008). Stearoyl-coenzyme A desaturase 1 gene expression increases after pioglitazone treatment and is associated with peroxisomal proliferator-activated receptor- responsiveness. *J. Clin. Endocrinol. Metab.* 93 4431–4439. 10.1210/jc.2008-0782 18697866 PMC2582575

[B26] Zheng G. Sayama K. Okubo T. Juneja L. R. Oguni I. (2004). Anti-obesity effects of three major components of green tea, catechins, caffeine and theanine, in mice. *In Vivo* 18 55–62. 15011752

